# Medical Society of the County of Erie

**Published:** 1911-11

**Authors:** 


### SOCIETY PROCEEDINGS

### Medical Society of the County of Erie

The Medical Society of the County of Erie, held a public meeting on October 16, 1911, at which Dr. Frederic C. Curtis spoke on Small Pox and Vaccination; and Dr. William A. Howe on Preventable Diseases. The former is Specialist in Dermatology, the latter Deputy Commissioner in the State Department of Health. The lectures were illustrated with stereopticon slides. The following representatives of the municipal and state departments of health discussed the subjects briefly: Drs. Francis E. Fronczak, William G. Bissell, W. S. Goodale, C. F. Durand, Edward Clark, Burt J. Maycock, John H. Pryor. The meeting was intended mainly for the education of the public, in view of the recent agitation against compulsory vaccination.

Dr. Fronczak made it very plain that he intended to enforce the law, both in letter and spirit, pointing out that people who believe the world to be flat, that the sun moves around the earth, and that vaccination does not protect against small pox must bow to competent authority. He considered the necessity of maintaining a municipal smallpox hospital a reflection on the intelligence of the city and dwelt upon the superiority of preventing smallpox, fires and the like, rather than of maintaining expensive plants for dealing with them when established. He mentioned the experience of Buffalo and other cities in dealing with small pox epidemics and quoted Heman Spaulding, Chief of the Chicago Bureau of Contagious Diseases and Chairman of the A. M. A. committee on methods of control of small pox, that unless a community is thoroughly protected by vaccination, it is impossible to stop an epidemic within two years at the best.

